# Multimorbidity clusters: translating research evidence into actionable interventions

**DOI:** 10.1007/s41999-025-01270-4

**Published:** 2025-07-11

**Authors:** A. Marengoni, F. Triolo, A. Zucchelli

**Affiliations:** 1 Aging Research Center, Department of Neurobiology, Care Sciences and Society, Karolinska Institutet and Stockholm University, Stockholm, Sweden; 2 Department of Clinical and Experimental Sciences, University of Brescia, Viale Europa 11, 25123 Brescia, IT Italy

**Keywords:** Multimorbidity, Cluster, Chronic diseases, Older people, Health care system

## Background

Multimorbidity, the co-existence of chronic diseases, becomes progressively more common with age, with more than 80% of adults over 60 years being multimorbid [1, 2]. About 30–40% of people with multimorbidity have both physical and mental health conditions, with multimorbidity being more prevalent in women and less wealthy individuals [1, 3, 4]. The burden faced by individuals with multiple chronic diseases derives from numerous treatments, fragmented care systems, and discrepancies between patients’ and doctors’ priorities, often enhanced by absent or conflicting clinical guidelines. As a result, the care of multimorbid patients is generally considered as qualitatively poor. Thus, a deeper understanding of the epidemiology of multimorbidity is necessary to develop interventions to prevent it, reduce its burden, and align health-care services more closely with patients’ needs.

No standard approach for the measurement of multimorbidity exists, but there is a rapidly growing body of literature attempting to study the structure defined by all co-occurring morbidities and to group them based on patterns. Different clinical conditions can be envisioned in a network, in which two or more of them are systematically associated if they share risk factors (e.g., genetics, lifestyles), pathological pathways, or drug-disease interactions [5, 6], if they cause one another, and/or if they affect overarching health outcomes such as function, cognition and quality of life, that are linked to other diseases [7–9]. However, despite the growing body of evidence, several questions remain about the applicability and implementation of a cluster medicine approach at the patient’s bedside, especially in primary care.

The aims of this perspective paper are (1) to summarize current literature on multimorbidity patterns, and the main statistical techniques used to identify clusters of diseases; (2) to suggest future research design and translation in clinical practice.

## Clinical relevance of multimorbidity patterns

Building on the need to characterize multimorbidity, cluster analysis techniques have emerged as a key tool to systematically identify patterns of co-occurring diseases. Since their introduction in the 1990s [10], statistical techniques for unsupervised data clustering have advanced technical capacity to perform complex clustering analyses; further, large population-based studies and electronic records databases have permitted to explore diseases’ clustering in different settings. A fresh preprint review quantified statistical methodologies used to evaluate clusters: hierarchical cluster analysis was the most used, followed by latent class analysis, K-center clustering and network-based approaches [11]. Based on rigorous statistical methods, numerous studies have examined the prevalence and characteristics of disease multimorbidity clusters across various populations (e.g., by ethnicity, country, or minority groups) [12–14], settings (e.g., community, primary care, or hospital) [15–17], and age groups [18–21].

Several systematic reviews have assessed the consistency of disease patterns across different settings and populations. In a systematic review of the literature, Prados Torres et al. found 63 patterns composed of three or more diseases and demonstrated relevant similarities for three groups of patterns. The first one comprised a combination of cardiovascular and metabolic diseases, the second one was related to mental health problems, and the third one with musculoskeletal disorders [22]. Busija et al. found that the two most replicable multimorbidity profiles among more than 400 were mental health and cardio-metabolic conditions [23]. In another recent review evaluating electronic health records in primary care, consistent patterns of multimorbidity emerged. Mental health and cardiovascular patterns were identified in all studies, often in combination with diseases of other organ systems (e.g. neurological, endocrine) [24]. Table 1 summaries findings from previous reviews on multimorbidity clusters; studies evaluating only dyads of diseases are not reported in the table, as well as studies focused on specific population groups (e.g., those affected by heart failure). Beyond the few clusters found to be consistently reported in previous studies, multimorbidity clusters tend to vary depending on populations, settings, age groups, socioeconomic status and other factors suggesting the need for individual-centered interventions [23]. Several studies have also identified a so-called ‘unspecific’ cluster including younger, healthier, less cognitively and functionally impaired people [25]. This group of individuals are affected by mild multimorbidity, but they are at risk of progression towards more complex multimorbidity patterns. In a longitudinal community-based study, during 12 years of follow-up, the main shifts among clusters involved participants in the unspecific cluster, who moved primarily to clusters characterized by cardiovascular, eye, respiratory, and musculoskeletal diseases [7]. Finally, a substantial body of literature has found specific clusters of diseases to be associated with higher risk of geriatric syndromes such as disability, frailty and dementia, as well as hospitalization and nursing home admission [26–29]. Specific clusters were also identified as risk factors for other conditions; cardiometabolic multimorbidity increases the risk of dementia [28], depression [30, 31], and kidney function decline [32]. Patterns characterized by psychological and cognitive disorders were consistently linked to a higher risk of falls in older people [33, 34].

**Table 1:** Summary of findings from previous reviews on multimorbidity clusters

First author and year of publication	Design, setting and cluster analysis methods	Number of studies included	Main findings
Prados Torres et al. 2014 [22]	Cross-sectional; general population, primary care and hospital; Agglomerative hierarchical cluster, Ratio observed/expected, multiple correspondence analysis, exploratory factor analysis, cluster analysis	14	Most replicable clusters were cardiovascular and metabolic diseases, mental health problems, and musculoskeletal disorders
Ng et al. 2018 [35]	Cross-sectional; general population and hospital; factor-analysis, hierarchical-clustering method, unified-clustering algorithm, multiple correspondence analysis, network and cluster analyses	41	Most replicable clusters were cardiovascular and metabolic diseases, mental health problems and allergic diseases
Busija et al. 2019 [23]	Cross-sectional; general population and outpatients; exploratory factor analysis, cluster analysis of diseases, cluster analysis of people, and latent class analysis	51	The two most replicable multimorbidity clusters were mental health conditions and cardio-metabolic conditions. Respiratory diseases, fractures and sensory impairments, as well as Parkinson’s disease and cognitive decline, were partially replicable
Rajoo et al. 2021 [13]	Cross-sectional; primary care and general population in Asia; exploratory factor analysis, observed/expected ratio, logistic regression, hierarchical cluster analysis	8	The most common multimorbidity patterns were “cardiovascular and metabolic diseases”, "mental health problems", "degenerative diseases", “pulmonary diseases," and "cancer diseases
Beridze et al. 2024 [24]	Cross-sectional and longitudinal; primary care electronic health record; multiple correspondence analysis, hierarchical cluster analysis, exploratory factor analysis, cluster analysis, principal component analysis, latent class analysis	16	Mental health and cardiovascular patterns were identified in all studies
Ioakeim-Skoufa et al. 2025 [36]	Longitudinal population-based studies; cluster analysis, markov models, and sequence analysis	10	Patients with Cardiometabolic Multimorbidity (CMM) develop additional cardiometabolic conditions or neurodegenerative and mental health disorders. Individuals from respiratory multimorbidity clusters often transition to CMM
Berner et al. 2025 [37]	Cross-sectional low- and middle-income countries; general population and primary care; exploratory factor analysis, latent class analysis	9	Most replicable clusters were Cardio-Metabolic, Musculoskeletal, Respiratory and Digestive/Visceral, Degenerative, and Mental Health

## Research design

Multimorbidity patterns have the potential to enhance clinical research by refining disease classification, enhancing treatment strategies, and improving risk prediction. Randomized clinical trials (RCTs) [38, 39] have shown mixed results in reducing the burden of multimorbidity or slowing the accumulation of chronic diseases, partly due to their focus on the simple presence of multiple morbidities, irrespective of their specific combination. Moreover, RCTs often exclude individuals with multimorbidity, limiting the possibility to test interventions in such population. To overcome these limitations, microsimulation models have emerged as promising instruments [40]. These are computational tools that simulate individual-level events based on probabilities, allowing for the in-depth exploration of complex systems and scenarios. They may provide a way to integrate evidence coming from multiple studies and data sources without the need to access the original raw data, thus mimicking disease progression in synthetic populations. Applied in the context of multimorbidity, microsimulation can help elucidate how individuals transition between different multimorbidity patterns over time and how these transitions relate to the development of adverse health outcomes such as disability, frailty or mortality. By accounting for diverse individual and contextual factors, these models can estimate the impact of different interventions on specific multimorbidity clusters. Microsimulation models will foster a more flexible, personalized and holistic approach to prevent multimorbidity, informing the next generation of intervention studies and policies. Complementary to this, research on multimorbidity biomarkers is expanding, potentially providing insights into the biological mechanisms driving MM development and progression [41, 42]. Such development could inform biomarker-driven strategies—particularly using blood-based (BB) and sensor-derives biomarkers—which hold promise for identifying shared pathophysiological pathways within patterns. These biomarkers may serve as dynamic indicators of disease onset, progression, and treatment response, potentially enabling clinicians to tailor interventions more precisely over time. Technologies like dried blood spot (DBS) sampling, which enable accurate and minimally invasive biomarker testing at scale, further support the feasibility of population-level monitoring. Taken together, this direction holds substantial promise for bringing multimorbidity clustering into clinical practice through biologically informed, scalable approaches to risk assessment, patient stratification, and ongoing care. Designing RCTs that specifically target clusters of chronic conditions could enable the identification of interventions tailored to patient subgroups sharing risk factors, disease trajectories, and health outcomes.

## Interpretation and translation

Despite the increasing availability of data and methods to evaluate disease clusters, it remains unclear whether identifying clusters of multimorbidity provides valuable information for everyday clinical practice. Advanced predictive algorithms incorporating disease clusters could enhance early risk identification, optimize clinical decision-making, and guide healthcare resource allocation. Yet, the clinical implementation of such prediction models presents several challenges. Potential criteria to ‘translate’ disease clusters findings to patients should be evaluated, e.g., clinically relevant diseases present in the cluster, clusters more prevalent in the population, clusters posing people at risk to develop dementia, disability or to shorten life expectancy. Potentially, several specific interventions could be adapted to different multimorbidity clusters; the unspecific cluster, for example, which includes younger, less cognitively and functionally impaired people at risk of developing other conditions and moving into more complex multimorbidity clusters during time could be a good target for primary and secondary prevention especially in primary care. For instance, recognizing cardiometabolic multimorbidity, which increases the risk of cognitive decline and dementia [28], may help target groups of vulnerable individuals amenable to specific interventions, such as physical activity and cognitive training. Further, people affected by specific patterns of multimorbidity may be expected to differ in their risk of developing functional decline and disability over time, another health outcome of critical importance in aging. In the Health and Retirement Study, subjects within a pattern comprising arthritis, hypertension and depression showed the highest level of functional limitations in daily living compared to healthy participants [43]. In a Swedish population compared to the Unspecific pattern, patterns characterized by varying degrees of clinical complexity were linked to increased risk of developing both basic and instrumental activities of daily living [26]. These findings underscore the potential of multimorbidity patterns to inform prioritization strategies in intervention planning, helping to target those most vulnerable to decline.

## Clinical impact

The exclusive reliance on a disease-by-disease approach to diagnosis, prognosis, and treatment has failed to catch the complexity of multimorbidity, resulting in suboptimal patient management. People with multiple conditions may need specialist care in an episodic manner, but their overall health care needs are likely to be best met by medical generalists who can integrate multiple sources of knowledge with individual needs assessment. This is increasingly the responsibility of primary care teams, which are known to provide more effective care for patients with multiple problems [44]. Indeed, findings on disease clusters challenge the single-disease framework by which most health care, medical research, and medical education are configured. The clustering of diseases is now seen as a key part of elucidating the complexity of multimorbidity. Understanding disease patterns may help reorient clinical training and practice toward a more integrated holistic model. This approach offers a heuristic for clinicians to prompt them to consider the co-existence of other diseases, or proactive interventions to prevent their development [45]. Further, the increased availability of data from primary care electronic health records over the last decade has provided researchers with access to large-scale data, potentially paving the way for a more comprehensive understanding of multimorbidity patterns in the general population. Indeed, given the longitudinal and generalist nature of the care provided by primary care physicians, such data sources would likely capture a broader spectrum of health conditions and healthcare utilization. In primary care, even subclinical stages of disease (i.e., impaired fasting glucose, mild cognitive impairment, pre-clinical hypertension, and slowed gait speed) could be investigated, which could challenge the artificial boundaries between diseased and non-diseased states. Further, drug prescriptions, as well as the social determinants of health, including education, occupation-life history, immigration status, and lifestyle behaviors could be assessed in relation to multimorbidity. Data from primary care may help to better elucidate disease etiology, and to determine whether the difference in disease patterns in different populations indicate differences in biological processes, environment or healthcare quality. Understanding multimorbidity clusters also provides an opportunity to better integrate patient perspectives into research and clinical care. Patient-reported experience measures (PREMs) and patient-reported outcome measures (PROMs) are likely to vary across multimorbidity clusters, reflecting differences in disease burden, treatment regimens, and quality-of-life concerns. Systematic evaluation of these measures could inform the development of cluster-specific endpoints that better align with patient needs, expectations, and lived experiences. This would be particularly relevant for advancing medicine strategies that take into consideration functional, psychological, and social well-being.

## Conclusions

In conclusion, over their life course, individuals accumulate multiple health conditions (both distinct and related) that often do not respect the current organization of medical research or practice. Each setting is characterized by specific multimorbidity patterns, which in turn are associated with diverse health outcomes. As such, by knowing the distribution of disease patterns clinicians can adopt more personalized, proactive care modes. This may also foster the development of new medical education programs that bridge multiple clinical specialties, health systems, pathological mechanisms, and biological systems and that holistically put patients as the central unit of concern.
